# Repertoires of SARS-CoV-2 epitopes targeted by antibodies vary according to severity of COVID-19

**DOI:** 10.1080/21505594.2022.2073025

**Published:** 2022-05-19

**Authors:** David J. Gregory, Augustin Vannier, Akiro H. Duey, Tyler J. Roady, Richard K. Dzeng, Maia N. Pavlovic, Michael H. Chapin, Sonia Mukherjee, Hannah Wilmot, Nic Chronos, Richelle C. Charles, Edward T. Ryan, Regina C. LaRocque, Tyler E. Miller, Wilfredo F. Garcia-Beltran, Julia C. Thierauf, A. John Iafrate, Steven Mullenbrock, Mark D. Stump, Randall K. Wetzel, Roberto D. Polakiewicz, Vivek Naranbhai, Mark C. Poznansky

**Affiliations:** a Vaccine and Immunotherapy Center, Massachusetts General Hospital, Boston, MA, USA; b Pediatric Infectious Disease, Massachusetts General Hospital, Boston, MA, USA; c Harvard Medical School, Boston, MA, USA; d Cardiology Care Clinics, Eatonton, GA, USA; e Division of Infectious Diseases, Massachusetts General Hospital Boston, Boston, MA, USA; f Department of Immunology and Infectious Diseases, Harvard T.H. Chan School of Public Health, Boston, MA, USA; g Department of Pathology, Massachusetts General Hospital, Boston, MA, USA; h Cell Signaling Technology, Danvers, MA, USA

**Keywords:** SARS-CoV-2, asymptomatic infection, seroprevalence, antibodies, epitopes, peptide array

## Abstract

Antibodies to SARS-CoV-2 are central to recovery and immunity from COVID-19. However, the relationship between disease severity and the repertoire of antibodies against specific SARS-CoV-2 epitopes an individual develops following exposure remains incompletely understood. Here, we studied seroprevalence of antibodies to specific SARS-CoV-2 and other betacoronavirus antigens in a well-annotated, community sample of convalescent and never-infected individuals obtained in August 2020. One hundred and twenty-four participants were classified into five groups: previously exposed but without evidence of infection, having no known exposure or evidence of infection, seroconverted without symptoms, previously diagnosed with symptomatic COVID-19, and recovered after hospitalization with COVID-19. Prevalence of IgGs specific to the following antigens was compared between the five groups: recombinant SARS-CoV-2 and betacoronavirus spike and nucleocapsid protein domains, peptides from a tiled array of 22-mers corresponding to the entire spike and nucleocapsid proteins, and peptides corresponding to predicted immunogenic regions from other proteins of SARS-CoV-2. Antibody abundance generally correlated positively with severity of prior illness. A number of specific immunogenic peptides and some that may be associated with milder illness or protection from symptomatic infection were identified. No convincing association was observed between antibodies to Receptor Binding Domain(s) (RBDs) of less pathogenic betacoronaviruses HKU1 or OC43 and COVID-19 severity. However, apparent cross-reaction with SARS-CoV RBD was evident and some predominantly asymptomatic individuals had antibodies to both MERS-CoV and SARS-CoV RBDs. Findings from this pilot study may inform development of diagnostics, vaccines, and therapeutic antibodies, and provide insight into viral pathogenic mechanisms.

## Introduction

The central role of antibodies in protection and recovery from COVID-19 has been a topic of intense study. IgGs to spike protein, and, in particular, neutralizing antibodies, have emerged as the strongest correlates of protection from severe disease in vaccinated people [1] and are associated with protection of convalescent COVID patients from re-infection [2]. Prophylactic administration of IgG results in protection in animal models [3–5] and monoclonal antibody therapy is associated with reduced hospitalization and death in some studies [6]. During primary infection with SARS-CoV-2, stronger and faster development of an IgG response is associated with reduced disease severity and faster recovery [7–9]. However, high titers are also found in patients who have severe or sustained COVID-19 [8,10], the clinical benefit of increasing antibody titers with convalescent plasma is uncertain [11] indicating that protection is not a simple correlate of antibody quantity. Additional factors may include timing of the response and qualitative differences between antibodies. Differences in IgG subtype and glycosylation have been found to associate with disease severity or time to recovery [12–14]. Here, we focus on how the specific repertoire of epitopes targeted in an individual may also influence clinical outcome. A number of studies have described the landscape of linear epitopes that are bound by antibodies [15–19]. These predominantly focus on hospitalized patients. Where association with different clinical outcomes is observed, individual epitopes are, generally, positively associated with disease severity, consistent with a stronger, more prolonged antigenic stimulus enabling development of antibodies against less immunogenic epitopes and/or epitope spreading during B cell maturation [15–18]. However, antibodies against some epitopes have been linked to less severe illness. For example, antibodies against epitopes in the fusion peptide and receptor-binding motif are associated with more rapid discharge from hospital and reduced ventilator use [20] or virus neutralization [21]. Other linear epitopes in nucleocapsid or spike are associated with increased survival or non-admission to ICU in hospitalized patients [18].

The potential protective role of preexisting antibodies developed following exposure to prior pathogens or other antigens has attracted interest. These have the potential to confer more rapid protection than antibodies developed in response to initial SARS-CoV-2 infection, including at initial exposure. Less pathogenic coronaviruses are endemic in human populations, normally causing symptoms of the common cold, and nearly all adults have detectable antibodies [17,22]. Recent infection with other coronaviruses is reported to speed recovery from COVID-19 [23,24]. However, this protection is likely to be transient, in line with other coronaviruses, where protection from infection with related species or strains wanes within 12 months [25]. Whether resulting from prior coronavirus infections or other exposures, antibodies that cross-react with SARS-CoV-2 epitopes are frequent in pre-pandemic serum and plasma samples [15,17]. However, the extent to which antibodies that cross-react against particular epitopes may protect from SARS-CoV-2 infection and COVID-19 severity is unclear.

We sought to further investigate the relationship between antibody repertoires and outcomes of infection, including in people who experienced no symptoms. We took advantage of a well-annotated sample of residents of a single city and its environs to link serological profiles with symptom and diagnosis history. We found no overall association between antibodies to the receptor binding domains (RBDs) of two less pathogenic seasonal betacoronaviruses and disease severity but were able to link specific epitopes of SARS-CoV-2 with both severe and mild outcomes of infection.

## Materials and methods

### Survey population

Participants were chosen from a larger cohort sampled over four days in August 2020. The study took place in a public square in the center of Chelsea, MA, a city in suburban Boston with a high immigrant and majority Hispanic population that suffered from particularly high infection rates during 2020 [26]. Care was taken in outreach to recruit a representative sample of local residents, including ensuring multilingual staff and literature. All participants were asked to fill in a questionnaire and provided blood and saliva for a battery of immunological and virological tests including clinically regulated quantitative PCR for active viral infection and presence of antibodies against SARS-CoV-2 nucleocapsid protein (Elecsys, Roche Diagnostics, Indianapolis, IN). Inclusion in this sub-study was determined by results of these tests and answers to the following survey questions:

1. Have you ever been diagnosed with COVID-19? (Yes/no – follow-up question requested date of PCR test)
2. Were you ever admitted to the hospital for COVID-19 infection? (Yes/no)
3. In the last 6 months, have you had any of the following COVID-19-associated symptoms? Please do not mention symptoms that you are sure you have because of another known underlying chronic condition. Check all that apply. (Fever/chills headache/muscle aches/reduced energy/runny nose/sore throat/coughing/chest pain/shortness of breath/recent loss of smell or taste/nausea or vomiting/abdominal pain diarrhea/skin rash or discoloration of fingers or toes/none)
4. Have you had **significant contact** with someone with COVID-19? (Yes/no)

Where more participants were enrolled than were needed for this sub-study, participants reporting ZIP codes corresponding to Chelsea and immediately neighboring communities were prioritized for inclusion. Sub-study participants were allotted into five groups, as shown in Table 1.

**Table 1.** Criteria used to determine group allocations

	Exposed	Negative	Asymptomatic	Mild	Hospitalized
*Test result*					
Current viral RNA	Negative	Negative	Negative	Negative	Negative
Current antibody	Negative	Negative	Positive	Positive	Positive
*Survey answer*					
Diagnosed	No	No	No	Yes	Yes
Hospitalized	No	No	No	No	Yes
Symptoms	None	None	None	Any	Any
Exposure	Yes	No	Any	Any	Any
*Number in category*					
Entire study	58	133	30	29	7
Included in this sub-study	30	29	29	29	7

The study was conducted in accordance with the Helsinki Declaration and applicable regulations and was approved in advance by Mass General Brigham Institutional Review Board (reference 2020P001081 and 2020P002274).

### RBD ELISA

Antibodies against multimeric recombinant Spike RBD proteins from five betacoronaviruses (SARS-CoV, SARS CoV-2, MERS-CoV, OC43, and HKU1) were detected by ELISA (Cell Signaling Technology). ELISAs were prepared using the following recombinant antigens (expressed in HEK 293 cells with 8 × His tags) coated onto 96-well plates: SARS-CoV-2 Spike RBD (multimeric, 319–591, Cell Signaling Technology #17862), SARS-CoV RBD (multimeric, 306–577), MERS-CoV RBD (multimeric, 364–655), OC43 Spike RBD (multimeric, 315–675), HKU1 Spike RBD (multimeric, 307–675). Plasma from the 124 participants was individually diluted 1:1000 (HKU1, MERS, and SARS-CoV) or 1:2000 (SARS-CoV-2 and OC43) using Sample Diluent A (Cell Signaling Technology #71637) and incubated separately on the antigen-coated ELISA plates for 1 h at 37°C and then washed. To recognize bound IgG, goat anti-Human IgG, Fc gamma Fragment Specific, HRP-Linked Antibody (Cell Signaling Technology #32935) was added for 30 min at 37°C. After washing, TMB Substrate (Cell Signaling Technology #7004) was added to develop color, and after addition of Stop solution (Cell Signaling Technology #7002), absorbance was measured at 450 nm. Each plasma sample was assayed in duplicate wells and the average used for subsequent analysis.

### Peptide and recombinant protein ELISA

One hundred and ninety-two peptides and recombinant proteins of SARS-CoV-2 and other human coronaviruses (Table 2) were adhered in duplicate wells of 384 well ELISA plate plates at a concentration of 2 µg/mL in PBS overnight at 4°C and blocked the following day. Plasma from each participant was assayed on a separate plate. In each case, plasma was diluted 1:500 with Sample Diluent A (Cell Signaling Technology 71637 Lot# 1) and incubated on the coated plate for 1 h at 37°C 1:500 dilution was chosen by using plasma from acutely ill, hospitalized patients (positive control) with plasma obtained in March 2020 from individuals with no evidence of exposure to SARS-CoV-2 (negative control). The plate was washed twice with PBS Tween (Cell Signaling Technology 9809S Lot#19) using a plate washer (Anthos Fluido 2). Fc gamma Fragment Specific, HRP linked anti-human IgG (Cell Signaling Technology 32935, Lot# 148044) was diluted 1:4000 with HRP diluent and (Cell Signaling Technology 13515 Lot# 179 & 182) added to the plate, which was then incubated at 37°C for 30 min. The plate was washed and wells incubated with TMB Substrate (Cell Signaling Technology 7004) at room temperature in the dark for 10 min to develop color. Stop Solution (Cell Signaling Technology 7002) was added and absorbance was measured at 450 nm and 650 nm using a plate reader (Molecular Devices, SpectraMax ABS Plus, SoftMax Pro 7.1).

**Table 2.** Antigens included in 384-well ELISA. Each well contained a single antigen with duplicate wells per antigen. Except where noted, recombinant proteins were expressed in HEK293 cells

Viral protein	Antigen
*Recombinant protein*	
SARS-CoV-2 Spike	Full-length, head, S1, S2, and RBD monomer
SARS-CoV-2 Nucleocapsid	Full-length (HEK293 expression) and 109–419 (bacterial expression)
SARS-CoV Spike	Head, RBD monomer
SARS-CoV Nucleocapsid	Full-length monomer
MERS-CoV Spike	S1 monomer
MERS-CoV Nucleocapsid	Full-length monomer
HKU1	RBD pentamer
*Peptide (all SARS-CoV-2)*	
Spike	Overlapping 22-mer array
Nucleocapsid	Overlapping 20-mer array
Envelope	1 × 12-mer
Membrane	3 × 12-mers
ORF1ab polypeptide	39 × 11-22-mers
ORF3b protein	1 × 22-mer
ORF8	3 × 14-24-mers

### Recombinant protein expression

Nucleocapsid proteins were secreted from Expi293 GnTI(-) cells and the Spike proteins and domains were isolated from HEK293 cells, except for the SARS-CoV-2 S1 and S2 monomer and HKU1 RBD pentamer, which were secreted from ExpiCHO cells. Culture conditions, including medium, were as recommended by ThermoFisher. The secreted proteins were purified by NiNTA column chromatography and eluted with 400 mM imidazole. Eluates were dialyzed against PBS pH7.4 and stored at 4°C. Quantitation was determined by absorbance at 280 nm.

### Statistical analyses

For the betacoronavirus RBD arrays, 450 nm absorbance values for each well were background corrected by subtracting 650 nm absorbance. Values from duplicate wells were averaged, then one-way ANOVAs were performed in GraphPad Prism version 9.2.0 for Windows to compare binding between groups.

450 nm absorbance values for 384-well ELISAs were background corrected in the same manner. Values for duplicate wells were averaged, then one-way ANOVAs performed using SciPy version 1.7.0. Outlier analysis was performed independently for each plate (plasma sample from an individual study participant). Outliers were defined as having A450 greater than 4 standard deviations from the mean of values in the plate. Antigens in the top 5% of A450 readings were excluded from mean and standard deviation calculations. The frequency with which each peptide antigen was determined an outlier within each group was expressed as a percentage of the samples within that group.

Logistic regression was performed with elastic net as the regularization penalty using the Caret package in R. Data was normalized and 4-fold cross validation with 100 repeats were used to train the model. Each population was classified against the remaining four populations.

## Results

### Study participants

Participants were grouped according to self-reported exposure history and current nucleocapsid antibody status, as described in Methods. The group characteristics are given in Table 3. Importantly, no participants had an active infection, as determined by detectable viral RNA, so the mild and hospitalized groups should be considered convalescent.

**Table 3.** Demographic characteristics of study participants

Group	Number of participants	Age (years, median, min – max)	% Female	Ethnicity (% Hispanic)
Exposed	30	37 (19–74)	57%	73%
Negative	29	43 (22–74)	53%	50%
Asymptomatic	29	46 (17–68)	47%	87%
Mild	29	47 (21–70)	55%	97%
Hospitalized	7	47 (18–58)	43%	100%
All participants	124	44 (17–74)	53%	78%

The difference in ethnicity (self-reported Hispanic/not Hispanic), especially between the negative and hospitalized groups, is suggestive and consistent with the known ethnic and socio-economic distribution of disease burden [27]. However, two other known risk factors addressed in the questionnaire, age and gender, were well balanced.

### Antibodies against RBDs of different betacoronaviruses

**Figure 1.**
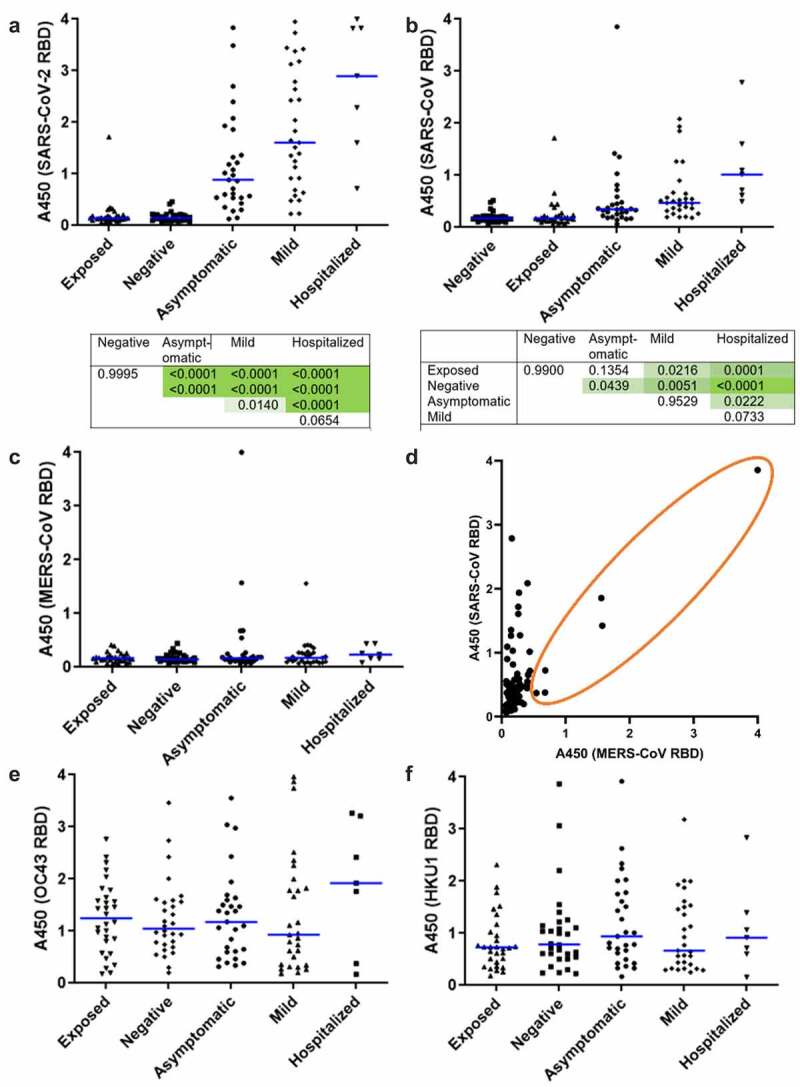
Prevalence of IgG specific for betacoronavirus RBDs in study cohort. Plasma was assayed by ELISA using recombinant, multimeric RBD as antigen. (a) SARS-COV-2 spike 319-591 with *p* values from ANOVA with Tukey HSD for all pairwise comparisons shown beneath. (b) SARS-CoV spike 384-655 with *p* values shown as above, (c) MERS spike 306-577. Each point indicates an individual participant. Blue bars show median values. N = 7–30. (d) Comparison of absorbance values for MERS and SARS-CoV RBD binding IgG for all participants. Each point indicates an individual participant, n = 124. The orange circle indicates participants with detectable amounts of MERS RBD-binding IgG, showing correlation with SARS-CoV-binding IgG in this subset. IgG specific to recombinant, multimeric RBD from seasonal betacoronaviruses, (e) OC43 spike 315-675, (f) HKU1 spike 307-675 assayed by ELISA as above.

To compare prevalence of antibodies against RBDs of related coronaviruses, we created a panel of recombinant proteins from five betacoronaviruses that cause disease of widely differing severity in humans. HKU1 and OC43 cause mild respiratory symptoms and are widely circulating, seasonal cold strains. 60–90% of adults have antibodies against these strains from prior infection [28,29]. In contrast, participants are very unlikely to have been exposed to highly pathogenic SARS-CoV and MERS-CoV and any antibodies are likely to be cross-reactive from other antigens. As shown in Figure 1, higher concentrations of antibodies against SARS-CoV-2 RBD were associated with greater severity of prior symptoms, with Exposed and Negative groups being indistinguishable. The difference between the two groups with prior diagnosed infection, Mild and Hospitalized, was also non-significant (*p* = 0.0654). This association is consistent with previous studies [10,14]. A similar trend is apparent in antibodies against RBD of SARS-CoV (Figure 1). This presumably results from antibodies produced following SARS-CoV-2 infection cross-reacting with the closely related SARS-CoV RBD. The trend was weak and non-significant for MERS-CoV (Figure 1), which is more distantly related to SARS-CoV-2, with greater sequence and structural divergence, and interacts with a different cellular target [30–32]. Interestingly, of the five donors with elevated α-MERS-CoV RBD, four were in the asymptomatic group (*p* = 0.0105, Fisher’s exact test). As shown in Figure 1, abundance of α-MERS-CoV RBD IgG in these individuals correlated with abundance of α-SARS-CoV RBD IgG, suggesting presence of broadly cross-reactive antibodies.

Abundance of IgG specific for RBDs of OC43 and HKU1 is shown in Figure 1, respectively. No relationship with SARS-CoV-2 infection and disease severity is apparent.

### Differences in abundance of antibodies against SARS-CoV-2 proteins between groups

To obtain a detailed assessment of the epitopes that are targeted by antibodies following natural infection and how these differ according to clinical severity, we constructed a 384-well ELISA with 192 different antigens. The antigens are described in Table 2 and include recombinant proteins and domains (some are the same as included in the panel of RBDs) and arrays of overlapping peptides that cover the complete SARS-CoV-2 nucleocapsid and spike proteins as well as predicted antigenic regions elsewhere in the proteome. Each well of the 384-well plate contained a single antigen, allowing plasma from an individual study participant to be assayed in duplicate on a single plate.

**Figure 2.**
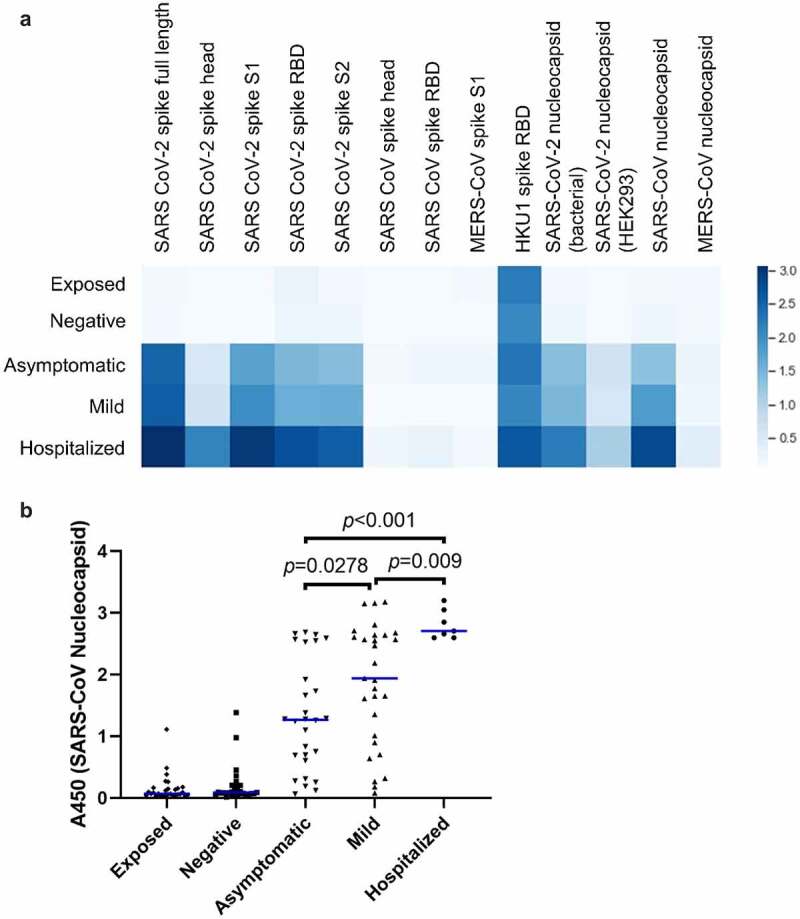
Prevalence of IgG specific for recombinant betacoronavirus proteins and domains. (a) Plasma was assayed by ELISA against the indicated recombinant protein antigens. The mean optical density seen for each group is shown as a heatmap, where darker color indicates greater average abundance of antibodies against the indicated antigen. (b) Abundance of antibodies to SARS-CoV nucleocapsid protein in individual donors. Each point indicates an individual donor. Blue lines indicate median values. Significant *p* values between the three infected groups are indicated (ANOVA with Tukey HSD, *p* < 0.05, n = 7–30). Pairwise differences are also significant between each of the three infected groups and each of two uninfected groups but are omitted for clarity.

Average abundance of antibodies against each of the protein antigens is shown in Figure 2. Significantly stronger binding was seen against all of the SARS-CoV-2 proteins in both diagnosed, recovered groups and participants who had undergone an asymptomatic infection when compared to those who had no evidence of infection. Average intensity increased with severity of symptoms. A similar pattern was seen for antibodies against the SARS-CoV nucleocapsid, which is consistent with the strong conservation between SARS-CoV and SARS-CoV-2 nucleocapsid sequences [33], but not spike proteins from other coronaviruses. Indeed, intensity of SARS-CoV nucleocapsid signal was able to distinguish accurately between each of the three infected groups in this assay (Figure 2). Interestingly, greater binding was seen to nucleocapsid 109–419 expressed in *E. coli* than to full-length (1–419) nucleocapsid expressed in HEK293 cells. This is consistent with extensive post-translational modification in mammalian hosts that may mask antibody epitopes [34].

### Differences in abundance of antibodies against SARS-CoV-2 peptides between groups

**Figure 3.**
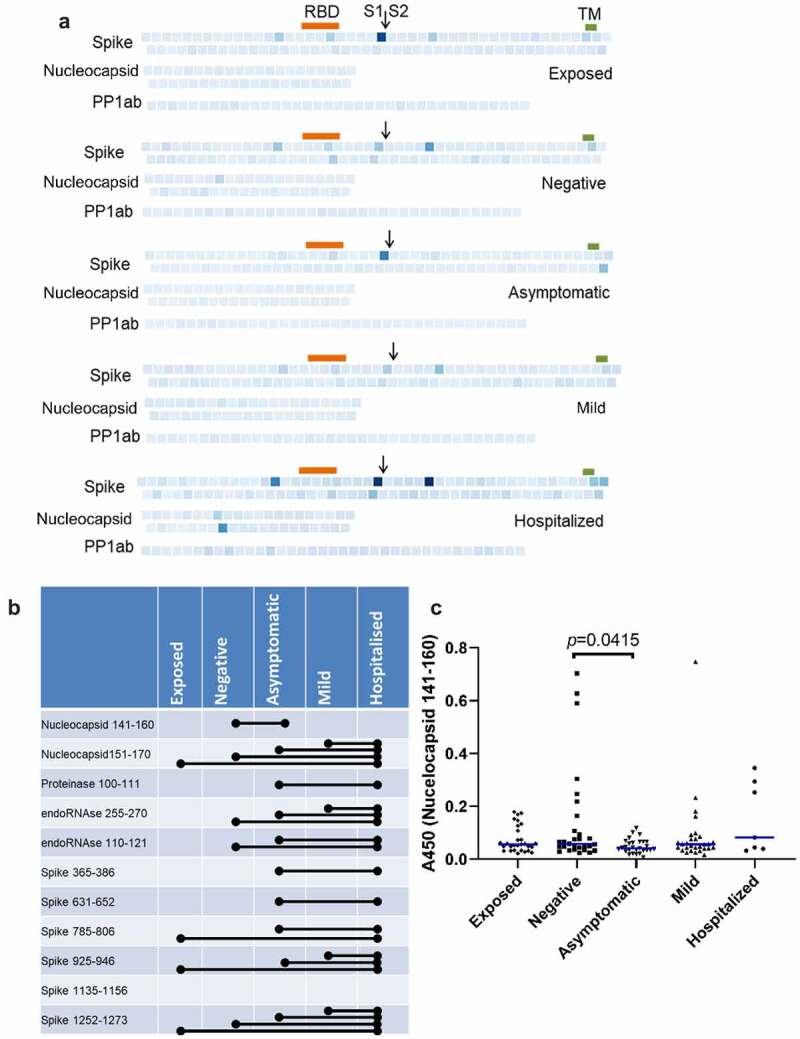
Prevalence of IgGs specific for SARS-CoV-2 peptides. (b) Individual peptides with significant variation between groups (*p* < 0.05, ANOVA, n = 7–30). Bars denote groups with significant differences by pairwise comparison (Tukey HSD, *p* < 0.05).

Figure 3 shows the average intensity of binding to each peptide in each group. Consistent with their smaller size, and so reduced presentation of epitopes, binding of antibodies to peptides was, generally, much weaker than to recombinant proteins. Significant pairwise comparisons between groups are shown schematically in Figure 3. Significantly greater binding was, generally, only seen in participants who had suffered severe disease resulting in hospitalization. The exception was Nucleocapsid 141–160, which is shown in detail in Figure 3. The presence of antibodies binding this epitope in participants with no evidence of exposure or infection may indicate recent infection with seasonal coronavirus. However, the absence of such antibodies in the exposed group does not support any inference that these antibodies are strongly protective against SARS-CoV-2 infection.

**Figure 4.**
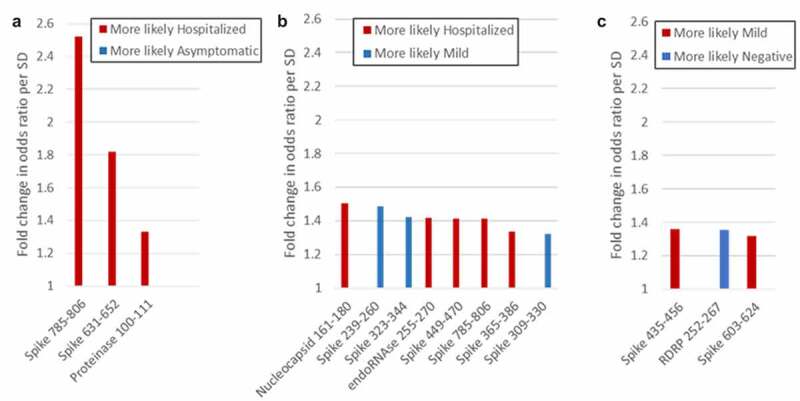
Peptides that differentiate between groups in logistic regression analysis. Peptides that distinguish between groups with more than 30% predicative effect above random are shown. (a) Hospitalized vs. asymptomatic; (b) Hospitalized vs. mild; (c) Mild vs. negative.

As a complementary approach, we used logistic regression to identify peptides that differentiate between groups in a pairwise comparison. Cross validation with varying mixing and regularization parameters indicated that model accuracies were acceptable for all pairwise comparisons except exposed-negative and asymptomatic-mild. As shown in Figure 4, binding to 10 peptides had a greater than 30% predictive effect in different pairwise comparisons. Of these, Spike 631–652, Spike 785–806, endoRNAse 255–270, and Proteinase 100–111 had also been identified by ANOVA. Of particular interest, RDRP 252–267, and Spike 239–260, 323–344, and 309–330 were all associated with less severe groups (negative over mild and mild over hospitalized, respectively).

**Figure 5.**
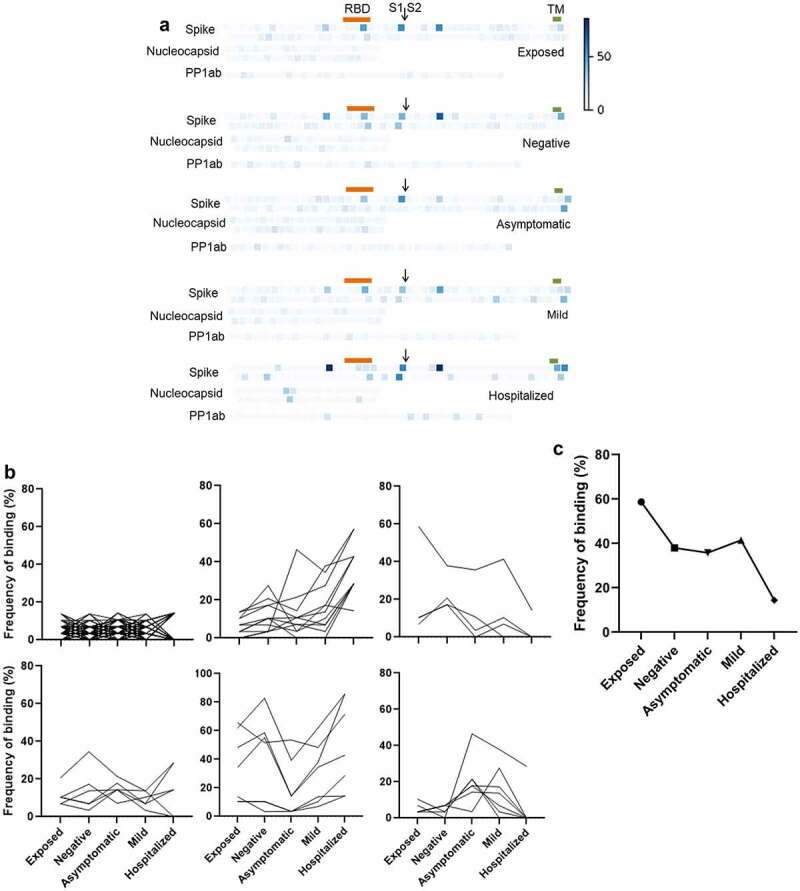
Frequency of seropositivity for IgGs specific to individual peptides in the different groups. (a) Frequency of seropositivity for each peptide. Each square indicates an individual peptide with color intensity proportionate to the frequency with which individuals in each group show antibody binding above background. Arrangement of squares indicates the approximate relative locations of the overlapping peptides in spike, nucleocapsid, and polyprotein 1ab proteins. Approximate locations of the RBD (319-541, orange), S1/S2 furin cleavage site (685, arrow), and transmembrane domain (1213-1237, TM, green) are shown for spike. (b) Distribution of peptides according to frequencies of seropositivity within each group. (c) Frequency of seropositivity for IgG specific to spike 506-525 in each group

The repertoire of specific epitopes targeted is highly heterogeneous between individuals. The lack of binding to a given epitope in some individuals reduces the sensitivity of analyses based on average intensities within each group. We therefore took a frequency approach, using outlier analysis to identify peptide epitopes that were targeted in each individual, and calculating the frequency with which each epitope was bound in each group. As shown in Figure 5, the frequency of binding of a particular epitope in a group ranged from 0% to 87%, but was generally low, as would be expected. Two peptides (Spike 645–666 and 785–806) were bound by a majority of individuals in all groups, consistent with prior reports of public epitopes [15]. Peptides could be differentiated into six categories according to how their frequency of binding varied between groups (Figure 5 and supplemental table). The large majority of peptides (147) were bound by antibodies in <15% of individuals in any group. Consistent with the linear regression and ANOVA results, the next largest category (11 peptides) showed increased binding in groups with increasingly severe infection. Of these, eight had also been identified by ANOVA and/or linear regression. Other categories showed positive or negative association with asymptomatic or mild disease, or no apparent difference between groups. Of particular interest, four peptides showed an inverse association with disease severity, with the lowest frequency of seronegative plasma being found in participants who had been hospitalized. The most pronounced of these was Spike 505–526, which is located in the RBD (Figure 5).

### Validation in an independent population

**Figure 6.**
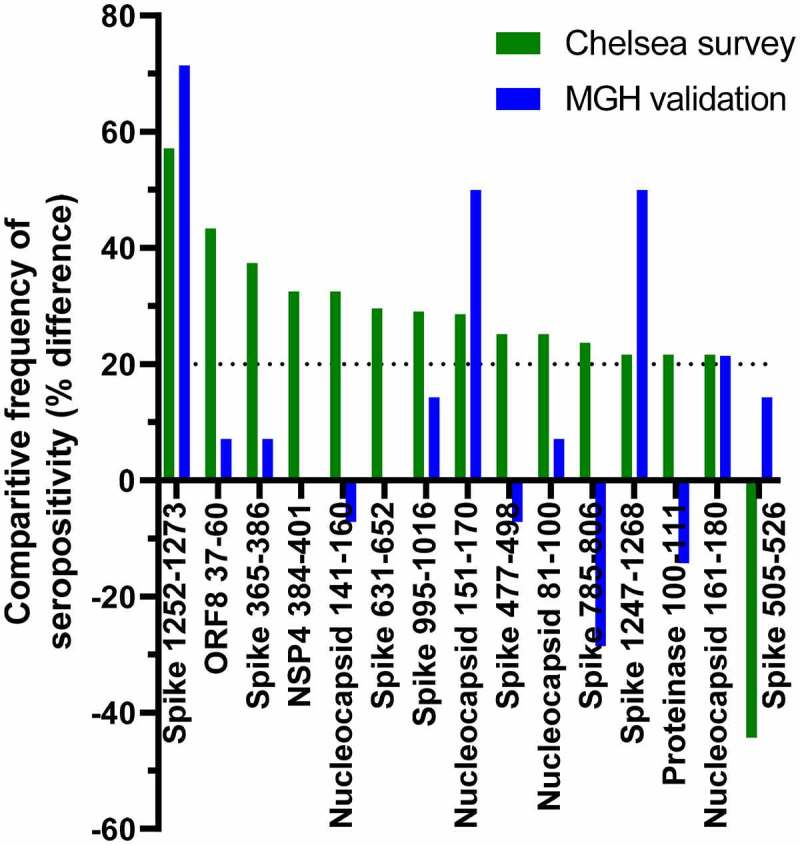
Comparison between peptide seropositivity frequencies in the primary dataset with an independent cohort. Peptides that showed >20 percentage point difference in frequency of seropositivity between participants in the Chelsea survey who had been hospitalized and those who reported exposure to SARS-CoV-2 are shown in green. The differences in frequency of seropositivity for IgG specific to those peptides between acutely hospitalized patients and people with no history of infection are shown in blue.

We sought to validate our findings in an independent cohort. Plasma samples from 14 volunteers without history of infection, including some with reported exposures, collected at Massachusetts General Hosptial and a site in Georgia were compared with heat-inactivated plasma from 14 patients hospitalized at MGH at the time of sampling, 6–19 days post-symptom onset, drawn during the early months of the pandemic. Because of the differences between sample sources and collection regimens, only the frequency analysis, in which optical densities are related to a sample-specific baseline, was attempted. As shown in Figure 6, of the 15 peptides that showed >20% point difference in frequency of binding between participants in the exposed, uninfected group of the original survey and those who had been hospitalized, 4 also showed large differences between the two validation groups. Significantly, these comprise two pairs of overlapping peptides, covering Spike 1252–1268 and Nucleocapsid 161–171, respectively. This strongly supports the finding that antibodies against specific epitopes within these regions develop during severe COVID.

## Discussion

In this study, we sought to relate the presence of antibodies against specific peptides of SARS-CoV-2 and against recombinant betacoronavirus proteins to clinical outcome following infection or exposure. We identified i) A strong correlation between severity of disease and abundance of antibodies against SARS-CoV-2 and, to a lesser extent, SARS-CoV antigens; ii) Specific immunogenic peptides; iii) Antibodies against some specific peptides that may be negatively associated with severe disease; iv) No convincing relationship between antibodies to RBDs from MERS or less pathogenic strains and SARS-CoV-2 infection or exposure and v) A small minority of individuals who appeared to have antibodies with broad specificity to RBDs of pathogenic betacoronaviruses following asymptomatic or mildly symptomatic infection.

The finding that individuals who have suffered most severe clinical disease following infection with SARS-CoV-2 exhibit the most pronounced antibody responses is consistent with prior reports. In these, people who have been hospitalized or admitted to ICU show the highest titers against recombinant SARS-CoV-2 proteins or domains, neutralizing antibody titers, or seroconversion against recombinant SARS-CoV-2 proteins, while those who have undergone asymptomatic infection have the lowest [35,36]. In addition to severity of disease, time since infection is known to affect overall antibody abundance [37]. While time from diagnosis to sample collection ranged up to seven months in the mild and hospitalized groups, it was not possible to ascertain time since infection in the asymptomatic group, so temporal aspects were not pursued here.

Prior studies of the relationship between specific peptide epitopes and disease severity have generally focused on more severely ill patients, for example, comparing ICU patients with those hospitalized on other wards, or patients who ultimately succumb to the disease with those who recover, but have also noted a trend toward increased signal following or during more severe disease [16–20]. Nucleocapsid 153–170 [16], Spike 613–638 [17] have previously been associated with more severe disease, which supports our findings. In apparent contrast, antibodies to Spike 451–465 have been observed more frequently in hospitalized patients than ICU patients [18] whereas antibodies to Spike 449–470 are associated with hospitalization over mild disease in our study. The contrasting associations with severity may indicate that the epitope is maximally immunogenic during severe illness (hospitalized) specifically but not during very severe (ICU) or mild illness. Alternatively, they may reflect the difference between active disease and more mature antibody profiles developed during convalescence, or may simply reflect the small sample size of both studies.

Our data particularly support the immunogenicity of two linear regions, and their association with severe disease. Nucleocapsid 161–171 and Spike 1252–1268 are each represented in two overlapping peptides that independently bind to IgG in plasma of study participants who had been hospitalized more frequently than participants in the exposed and negative groups. Furthermore, in our validation dataset, both of these pairs of peptides also bind IgG in plasma of acutely ill, hospitalized patients more frequently than in plasma of healthy individuals without evidence of infection. Since both sample sets are small and, as described below, there are limitations to the applicability of the validation sample set, the identification of all four peptides in both sample sets in spite of these differences indicates a robust observation. Nucleocapsid 161–171 is further supported by data from other studies [16,38]. This epitope can therefore be considered highly immunogenic and may be valuable diagnostically. Nucleocapsid 161–171 lies within a region that associates with antiviral activity of convalescent plasma *in vitro*, but overall, the association with severe disease suggests that the effectiveness of antibodies against these epitopes at protecting against COVID-19 is limited. Interestingly, Spike 1252–1268 lies within the cytoplasmic tail of spike, so is unlikely to be exposed to extracellular antibodies in either viral particles or infected cells.

A primary goal of this study was to identify linear B cell epitopes that are associated with reduced symptoms or no infection after exposure, either because antibodies that bind them are directly protective or because they are biomarkers of an effective broader immune response. The fact these samples were obtained some months into the pandemic and after recovery from any reported infection events means that it is difficult to differentiate between antibodies that developed in response to SARS-CoV-2 infection and were associated with an effective and targeted immune response, or that pre-dated the infection and had been beneficially cross-reactive against SARS-CoV-2. Nonetheless, the epitopes identified here may be of particular value in design of further vaccines and antibody therapies.

This study has employed an exceptionally well-annotated sample of an urban population (Chelsea, MA), which has enabled robust and detailed comparison between classes of infection severity. However, confidence in conclusions is limited by the size of the groups. This is particularly true for the exposed but uninfected group, where numerous factors aside from antibody repertoire may lead to no infection. Nonetheless, the reduced seropositivity toward Spike 506–525 among study participants who had been hospitalized relative to other groups, especially the exposed, is at least consistent with antibodies targeting this epitope, which are present in about 40% of the study population, being protective against severe COVID-19 and/or infection with SARS-CoV-2. The observation was not repeated in our small-scale validation study, instead the reverse was seen, but this contrast should be treated with caution. In addition to the similarly small sample size, the sample set was less well matched than the primary, Chelsea samples. For safety reasons, the acute patient plasma had been heat treated. Unlike the primary sample set, where the groups all showed similar age distributions, it is likely that the actively hospitalized patients were older than the healthy controls. Finally, the patient samples were taken between 12 and 19 days after symptom onset and while the patients were undergoing intensive therapeutic interventions. These actively developing antibody responses may not be comparable to the more mature profiles seen in convalescent individuals and may be confounded by the effects of treatments. Thus, while it is at least encouraging where both analyses reach the same conclusions despite the technical differences, contrasts between them should be treated with caution.

Seropositivity to other peptides, such as Spike 1009–1030, is only seen or strongly biased toward people who were infected without symptoms; antibodies against these epitopes may therefore be associated with protection. Our validation study did not include samples from people who had no or mild symptoms, so an adequately powered study addressing these epitopes in particular would be especially valuable.

Samples were collected in August 2020, prior to documented spread of the variants of concern, raising the question of whether mutations in the variants will affect the affinities of antibodies elicited by developed in response to infection with the earlier strain. Of the 21 immunogenic peptides in spike identified here, responses to Spike 981–1002 and Spike 1009–1030 may be affected by mutations S982A and D1118 H in the alpha variant. Beta mutations Δ242–244 and R246Y may be affect recognition of Spike 239–260, and Spike 435–456 and Spike 449–470, which form an overlapping pair, may be affected by L452 R reported in Delta. Omicron contains more reported mutations that lie within the immunogenic spike peptides identified here, consistent with the omicron mutations both being more numerous and being associated with immune escape. Interestingly, none of the immunogenic peptides from nucleocapsid are affected by mutations in any variants of concern, including omicron. While the effects of mutations on specific and polyclonal neutralizing antibodies is well documented [39,40], it remains to be established whether current and future mutations are driven by immune pressure as opposed to receptor interaction or other influences. If protection by antibodies against the peptides reported here is or becomes significant at the population level, we might predict that mutations in future variants will affect these peptides.

Our data do not show any significant relationship between infection or exposure history on abundance of antibodies that bind RBDs from two widely circulating species of betacoronaviruses that infect humans with lower pathogenicity. While previous reports have given varying results, the current consensus is that recent infection with an endemic seasonal coronavirus, resulting in higher antibody titers, reduces COVID-19 severity [23,24], and that, conversely, SARS-CoV-2 can boost titers of antibodies against endemic seasonal strains [41,42]. In a post-exposure survey, such as this one, these effects may counteract each other to some extent. However, these effects rely on sequence and structural homology, and studies reporting the strongest effects have studied entire recombinant spike proteins or the c-terminal domain. Like others who have reported minimal cross-reactivity [43], our assay tested antibodies specific to the RBD, which is less well conserved, so might be expected to elicit species-specific responses.

Taken together, our data show relationships between specific peptide sequences and outcomes of prior infections and exposures to SARS-CoV-2. Peptides associated with less severe illness may be especially valuable targets for vaccine and recombinant antibody therapies, and provide insight into viral pathogenic mechanisms. Insights from this pilot study merit confirmation and expansion in larger scale screens.

## Supplementary Material

Supplemental Material

## Data Availability

The authors confirm that the data supporting the findings of this study are available within the article and its supplementary materials.
